# The molecular mechanisms underlying diabetic complications

**DOI:** 10.1186/1687-9856-2013-S1-O1

**Published:** 2013-10-03

**Authors:** Francesco Chiarelli, M Loredana Marcovecchio

**Affiliations:** 1 Department of Paediatrics, University of Chieti, Chieti, Italy

## 

Diabetes mellitus is one of the most common chronic diseases worldwide and is associated with an increased morbidity and mortality. Diabetes is characterized by chronic hyperglycemia and alterations of cellular homeostasis, which lead to diffuse vascular damage.

The microvascular complications of diabetes, resulting from a damage of the microvasculature of the kidney, retina and neurons, include retinopathy, nephropathy and neuropathy. As a consequence of microvascular pathology, diabetes is an important determinant of blindness, end-stage renal disease and a variety of debilitating neuropathies. In addition, diabetes is associated with cardiovascular disease, which is an important contributor to the overall morbidity and mortality associated with this condition.

Several risk factors are implicated in the pathogenesis of diabetic complications and they can be either modifiable (glycemic control, hypertension, dyslipidemia, diet, smoking) or non-modifiable (diabetes duration, age at onset, puberty, genes).

The pathogenesis of diabetic vascular complications is complex, with the involvement of several mechanisms and a clear contribution of genetic factors.

Hyperglycemia is a key determinant of vascular complications of diabetes and there is extensive evidence showing that both acute and chronic hyperglycemia has a deleterious effect. Hyperglycemia contributes to the development of vascular complications through several mechanisms: activation of the polyol and hexosamine pathways, activation of protein kinase C, increased oxidative stress, increased production of advanced glycation end-products, increased synthesis of growth factors, cytokines and angiotensin II. These factors can, in turn, induce a diffuse endothelial dysfunction and contribute to the progressive development of micro- and macrovascular complications and multiorgan damage. Growing evidence suggests that increased oxidative stress, induced by several hyperglycemia-activated pathways, is a key factor in the pathogenesis of endothelial dysfunction and vascular disease. Several mithochondrial and other intracellular pathways are implicated in the increased production of oxidants, which is often associated with reduced antioxidant defences. In addition, recent studies suggest the involvement of epigenetic mechanisms as well as of microRNAs in the pathogenesis of diabetic complications.

The understanding and characterization of the molecular mechanisms underlying the development of vascular complications of diabetes is of paramount importance as this could help the development of better preventive and treatment strategies.
